# C-Jun N-terminal kinase (JNK) isoforms play differing roles in otitis media

**DOI:** 10.1186/s12865-014-0046-z

**Published:** 2014-10-14

**Authors:** William Yao, Meredith Frie, Jeffrey Pan, Kwang Pak, Nicholas Webster, Stephen I Wasserman, Allen F Ryan

**Affiliations:** Departments of Biology, University of California, San Diego, La Jolla, CA 92093 USA; Departments of Surgery/Otolaryngology, University of California, San Diego, La Jolla, CA 92093 USA; Research Service, Veterans Administration Medical Center, San Diego, CA 92151 USA; Departments of Medicine Endocrinology and Metabolism, University of California, San Diego, La Jolla, CA 92093 USA; Departments of Medicine/Allergy & Immunology, University of California, San Diego, La Jolla, CA 92093 USA; Surgery/Neuroscience, 0626, UCSD School of Medicine, 9500 Gilman Drive, La Jolla, CA 92093-0626 USA

**Keywords:** Middle ear, Nontypeable haemophilus influenzae, Infection, MAP kinase signaling, Tissue proliferation, Inflammation

## Abstract

**Background:**

Innate immunity and tissue proliferation play important roles in otitis media (OM), the most common disease of childhood. CJUN terminal kinase (JNK) is potentially involved in both processes.

**Results:**

Genes involved in both innate immune and growth factor activation of JNK are upregulated during OM, while expression of both positive and negative JNK regulatory genes is altered. When compared to wildtypes (WTs), C57BL/6 mice deficient in JNK1 exhibit enhanced mucosal thickening, with delayed recovery, enhanced neutrophil recruitment early in OM, and delayed bacterial clearance. In contrast, JNK2^−/−^ mice exhibit delayed mucosal hyperplasia that eventually exceeds that of WTs and is slow to recover, delayed recruitment of neutrophils, and failure of bacterial clearance.

**Conclusions:**

The results suggest that JNK1 and JNK2 play primarily opposing roles in mucosal hyperplasia and neutrophil recruitment early in OM. However, both isoforms are required for the normal resolution of middle ear infection.

**Electronic supplementary material:**

The online version of this article (doi:10.1186/s12865-014-0046-z) contains supplementary material, which is available to authorized users.

## Background

Otitis media (OM) is one of the most prevalent and costly acute medical problems that afflict children and infants aging from six months to six years [1-3]. Although acute OM may not cause immediate concern in developed countries, persistent and chronic OM produces a sustained conductive hearing loss and may result in irreversible damage to the middle and/or inner ear. In the developing world, in contrast, OM causes an estimated 30,000 deaths per year, and contributes to half of the world’s burden of serious hearing loss [4]. Understanding the pathogenesis of OM is thus an important research goal.

Consequences of OM to the middle ear (ME) include hyperplasia of the ME mucosa with the potential for mucus secretion, leukocytic infiltration into the ME cavity, and ME effusion; ME inflammation associated with OM can also cause tissue damage and intense pain. While OM is multifactorial, one of its most prominent causes is bacterial infection. In particular, as the usage of pneumococcal vaccines has increased, the incidence of OM caused by nontypeable *Haemophilus influenzae* (NTHi) has risen [5]. The mechanisms by which bacteria influence the ME during OM is a subject of increasing interest. With respect to the context of the present study, there is extensive evidence that innate immune receptors play a role in the resolution of acute OM e.g. [6], but the downstream effectors of pathogen-induced responses in the ME are not well understood. Both the Toll-like receptor (TLR) and NOD-like receptor families of innate immune receptors can signal via CJUN N-terminal kinase (JNK) to activate proinflammatory target gene expression including cytokines and chemokines. However, JNK is also the product of a proto-oncogene, and like other mitogen activated protein kinases (MAPKs) [7] can be a potent mediator of tissue growth [8]. Finally, JNK is a stress protein and has been shown to mediate apoptosis in some circumstances [9].

Elucidating JNK signaling pathways activated during OM would not only improve our understanding of ME immune signaling and its regulation, but might also provide new gene targets for alternative OM treatments. JNK signaling is complex, with numerous upstream and downstream molecules potentially participating. TLR signaling via JNK involves the TLR adaptor MyD88, which activates IRAK4 and TRAF6, in turn activating TAK1. TAK1 phosphorylates the MAP kinase kinases (MAP2Ks) 4 and 7, which in turn activate JNK [10]. Activated JNK phosphorylates members of the JUN family of proteins, including CJUN, JUNB, JUND, as well as ATF2. Phosphorylated CJUN homodimerizes or forms heterdimers with phosphorylated FOS or ATF2, to form the AP-1 transcriptional activator, which can potentially regulate many genes [11,12]. Growth factor binding to their cognate receptors recruit adaptors, including SHC, GRB and SOS, can activate the small GTPases KRAS, and CDC42 [13], which in turn activate MAPK kinase kinase kinases (MAP3Ks) 1 and 4. These converge with TLR signaling by activating MAP2Ks 4 and 7 to, in turn, phosphorylate JNK.

Activation of the JNK pathway is also regulated by multiple scaffolding proteins, which function to collect signaling effectors into molecular complexes to regulate signal transduction and localize the complexes to specific parts of the cell. These include the JNK-interacting proteins (JIPs) 1–3, which can aggregate MLK, MAPKs 2 and 7, and JNK to enhance JNK activation. Similarly, POSH (plenty of SH3) interacts with MLK3 to aid in JNK phosphorylation [14] while arrestin β2 (ARRB2) has been shown to dock directly to MAP2K4 [15] and activate JNK [16]. The dual specificity phosphatase (DUSP) proteins are scaffolds that influence MAPKs with varying actions and specificity [17]. DUSP1, 2, 8 and 10 positively regulate JNK. DUSP 16 [18], 18, 19 (SKRP1) and 22 inhibit JNK activity [19,20]. CDC42EP2 can also inhibit JNK by blocking the activity of upstream CDC42 [21], while the JNK target CJUN can be inhibited by competition with JUNB.

JNK signaling is complicated by the fact that JNK is not one protein. Three JNK genes (*Jnk1, Jnk2, Jnk3*) can be alternatively spliced into 10 isoforms, which allows for different JNK activities in specific tissue types [22]. The *Jnk1* and *Jnk2* genes are expressed in tissues throughout the body, but *Jnk3* has expression limited primarily to the brain, heart and testes [7]. JNK1 and JNK2 have been implicated as important in immune signaling and evidence has shown that JNK1 and JNK2 protein activation is increased in rat ME mucosa during OM [23].

The JNK1 and JNK2 isoforms have been shown to perform different signaling roles to induce specific physiological changes [22]. However, differences between JNK1 and JNK2 actions are not well characterized, and most JNK effectors are stimulated by both JNK1 and JNK2, making it difficult to determine signaling differences between the two molecules.

The objective of this study was to investigate JNK signaling networks during OM, by documenting the expression of various genes in the JNK signaling cascade during the course of a ME infection, and by comparing OM in mice deficient in JNK1 or JNK2 with that in wildtype (WT) mice. We hypothesized that genes encoding many elements of JNK signaling pathways would be regulated during OM, and that JNK1 and JNK2 would contribute to different aspects of the OM phenotype.

## Methods

### Animals

Experimental methods and protocols were performed on healthy, naive 60–90 day old (~25 gm) mice, according to the National Institutes of Health guidelines on the care and use of laboratory animals and approved by the Institutional Animal Care and Use Committee of the San Diego VA Medical Center. Mice were used since acute OM in this species is quite similar to the disease in humans [3,24], and since gene knockouts are available. Animals were housed under SPF conditions with 4–6 mice per cage, free access to food and water, and environmental enrichment. Gene array studies were performed on C57/CB F1 hybrid mice (Jackson Labs, Bar Harbor, ME), to reduce the influence of the recessive mutations common in inbred mouse strains. Knockout experiments were performed on mice systemically deficient in JNK1 or JNK2 on a C57BL/6 background, and upon C57BL/6 WT controls (bred in-house from Jackson Labs stock), since they were produced on this background. Gene deficient animals are most frequently created in this species/strain. Generating C57/CB F1 hybrids with the KOs would not have been possible. It should be noted that we have performed arrays on C57BL/6 mice at 6 and 48 hours after NTHi inoculation, and the results are quite similar to those seen with C57/CB F1 hybrid mice.

### Bacterial strain and culture conditions

Minimally passaged *Haemophilus influenzae* strain 3655 (nontypeable, biotype II; extracted from the ME of a child with OM) was grown, inoculated, and used at a concentration of 10^5^ -10^6^ bacteria/ml to generate an inflammatory response within the ME. Inocula were prepared as described by Melhus and Ryan [3].

### Surgical procedure

Surgeries were performed aseptically in a designated area of the laboratory. WT, JNK1^−/−^, and JNK2^−/−^ mice were anesthetized with an intraperitoneal injection of 0.1-0.2 ml per 30 g bodyweight of rodent cocktail (13.3 mg/ml ketamine HCL, 1.3 mg/ml xylazine and 0.25 mg/ml acepromazine), which provides brief, deep anesthesia. MEs were exposed bilaterally by a ventral approach through a midline neck incision. ME bullae were then punctured using a 25-gauge needle. Approximately 5 microliters of NTHi (500–5000 cfu at 10^5^ -10^6^ bacteria/ml) were injected into the ME cavity using a 30-gauge needle, which allowed air to exit the ME opening during injection. Excess fluid was soaked up with a sterile cotton swab. The wound was closed and the skin incision was stapled. The mice were injected with the analgesic buprenorphine and lactated Ringer’s solution subcutaneously. Mice were examined for fluid leakage into the external ear canal to ensure no penetration of the tympanic membrane had occurred from the 25-gauge needle. Surgeries were timed to permit sacrifice at the appropriate interval. All animals were monitored after surgery until ambulatory, and then daily for health status until sacrifice. No adverse events occurred.

### DNA microarray

Expression of genes involved in JNK signaling was evaluated during the course of an acute episode of OM in WT mice by DNA microarray. For each time point evaluated, twenty WT mice were inoculated with NTHi as above. The ME mucosae were harvested at each of the following intervals: 0, 3 and 6 hours, as well as 1, 2, 3, 5 and 7 days after NTHi inoculation. 0 hour (control) mice received no treatment. The tissue was homogenized in TRIzol™ (Invitrogen, Carlsbad, CA) and total RNA was extracted. RNA was labeled and hybridized to two Affymetrix MU430 2.0 microarrays. This procedure was then duplicated with an additional 20 mice for each time point, to obtain an independent replication. Gene transcript expression levels were evaluated using variance-modeled posterior inference (VAMPIRE) [25]. Functional gene families were assessed by gene ontogeny (GO) analysis, and specific genes were assessed at individual time points, after Bonferonni correction for multiple tests, using Genespring GX 7.3 (Agilent). Individual gene behavior was evaluated as fold change from expression observed in uninoculated (0 hour) control mice.

As a control, mock injection of only saline was performed in identical groups of mice. This also resulted in the significant regulation of some genes. However, they almost invariably exhibited lower levels of regulation than that seen after NTHi inoculation. For the 47 genes reported here, 30 were not regulated by saline injection, and all of the remaining genes were regulated at lower levels, many substantially so (data not shown).

### Histology

OM was evaluated in 3 experimental groups: 33 JNK1^−/−^ mice, 33 JNK2^−/−^ mice, and 33 WT control mice with the same genetic background (C57BL/6). JNK knockout and C57BL/6 strains were obtained from Jackson Labs. Each of these groups was randomly subdivided into sets of 3 mice, 1 set for each of 11 time points. When more than one batch of bacteria was used each batch was distributed between all groups. The mice were bilaterally inoculated in the ME with NTHi, and were sacrificed under general anesthesia by intracardiac perfusion with 3 ml of PBS followed by 3 ml of a 4% paraformaldehyde solution at 0, 6 or 12 hrs or 1, 2, 3, 5, 7, 10,14, or 21 days after inoculation. In all experiments, the 0 hr time point represents untreated MEs. The MEs were extracted, postfixed with 4% PFA overnight, and decalcified in an 8% EDTA/4% PFA solution over a 14 day period. The ME bullae were embedded in paraffin and serially sectioned at 7 micrometers. Sections were stained with hematoxylin-eosin and mounted. Images of the ME cavity were digitally recorded and mucosal thickness and percentage area of the ME lumen that contained inflammatory cells were determined from standardized areas spaced throughout the ME, using 6 MEs per condition, as described previously [26].

To identify the cells present in the ME, high-magnification (400×) images were captured for regions with inflammatory cells in the lumen. The number of neutrophils and macrophages present in each 400× image was counted, and counts from 3 images were averaged for each ME. Data from 6 MEs were evaluated per condition.

### Bacterial clearance

The presence of live bacteria in the ME was evaluated in 3 experimental groups: 21 JNK1^−/−^ mice, 21 JNK2^−/−^ mice, and 21 WT control mice (C57BL/6). Each group was randomly subdivided into sets of 3 mice, 1 for each of 7 time points. MEs were extracted from 3 WT and 3 JNK knockouts for each isoform (JNK1 or JNK2) at 1, 2, 3, 5, 10, 14, and 21 days after ME inoculation. The MEs were opened and a 1-microliter loop was used to obtain a sample from the ME lumen for NTHi culture onto chocolate agar plates with nutrients specific for NTHi growth. For MEs without fluid present, the loop was rubbed across the ME mucosal surface. Each loop was T-streaked onto 4 successive quadrants of each plate. The plates were then analyzed after 24 hours’ incubation at 37°C. The identity of NTHi was verified by plate specificity and by colony color and morphology.

Bacterial data were analyzed by determining the presence or absence of NTHi in each plate, and quantified by counting the number of quadrants in which colonies were observed.

### Statistical analysis

Data were analyzed using Statview (Scientific Computing, Cary, NC). Parametric data were analyzed by ANOVA with the Fisher post-hoc test, Bonferroni corrected for multiple comparisons, for tests of individual time points. Leukocyte counts were measured as percentages, and these data were analyzed with the Kruskal-Wallace nonparametric ANOVA equivalent, and the Mann–Whitney *U* test for individual time point analysis. All animals in each group were included in the analyses, and no animals were excluded.

## Results

### Expression of JNK signaling genes in the ME during OM

**Figure 1:**
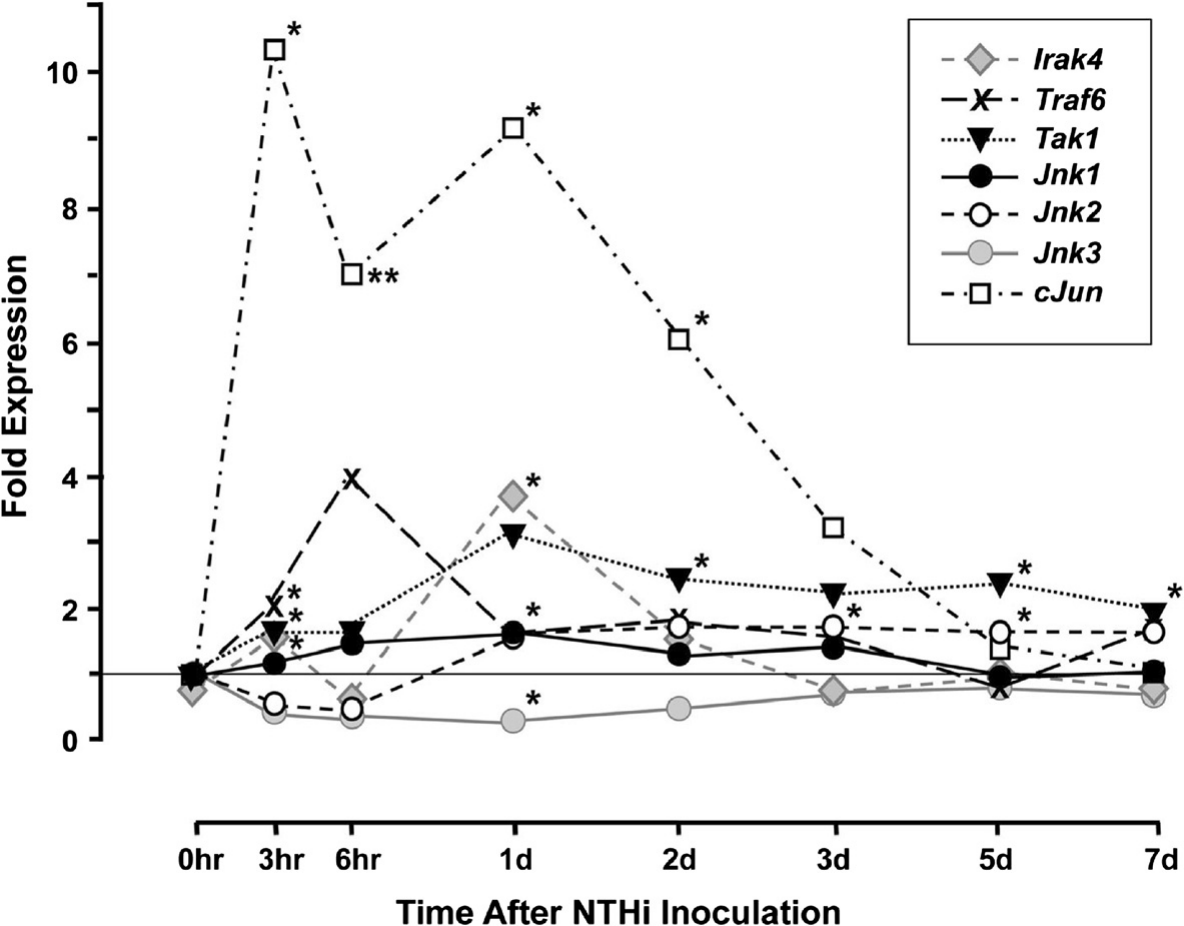
**Regulation of JNK signaling genes during OM.** ME expression of genes related to the JNK signaling pathway in WT mice during OM, assessed by DNA microarray. Genes involved in TLR JNK signaling.

**Figure 2:**
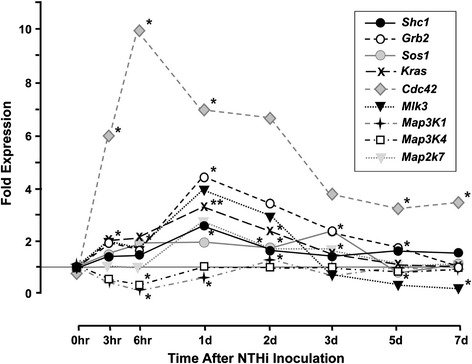
**Regulation of JNK signaling genes during OM.** ME expression of genes related to the JNK signaling pathway in WT mice during OM, assessed by DNA microarray. Genes invoved in growth factor JNK signaling.

**Figure 3:**
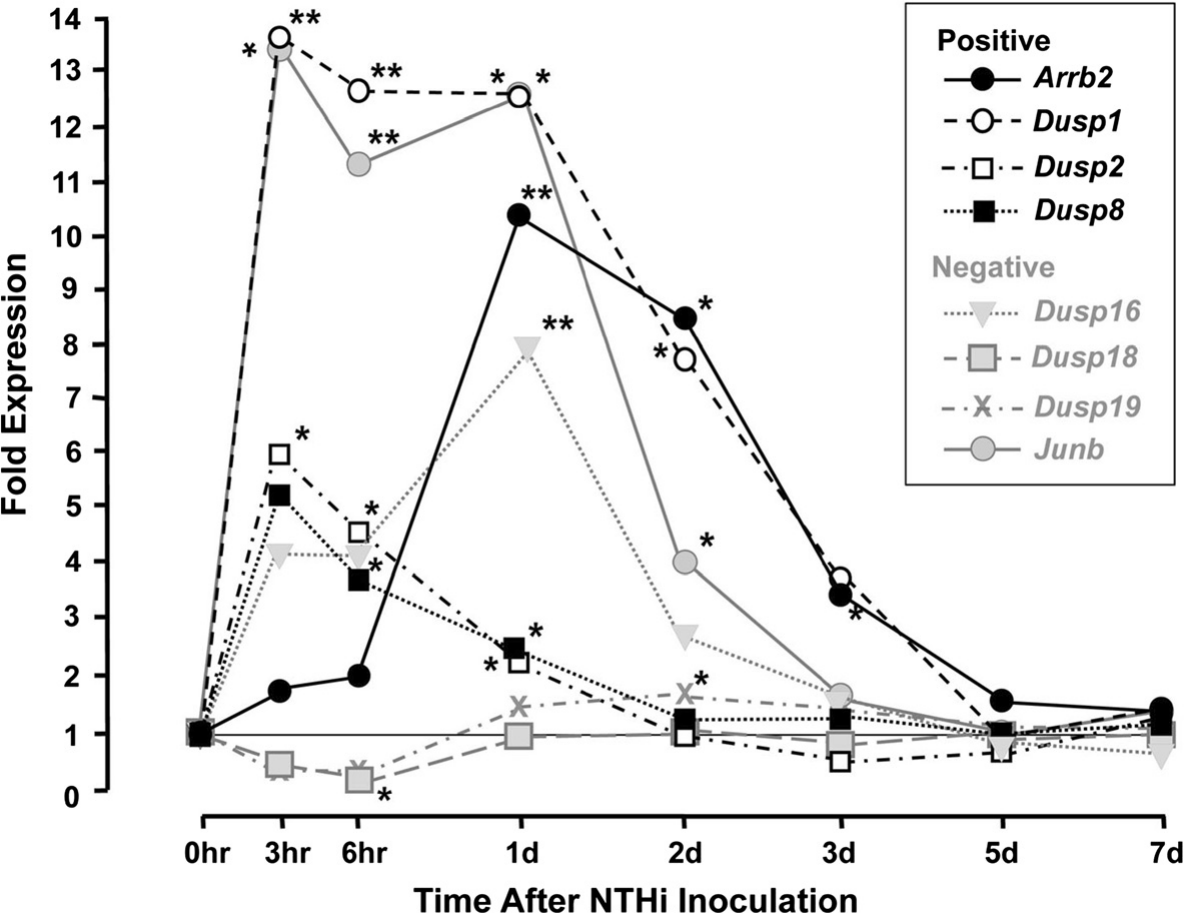
**Regulation of JNK signaling genes during OM.** ME expression of genes related to the JNK signaling pathway in WT mice during OM, assessed by DNA microarray. Genes involved in JNK signaling regulation. The genes encoding JNK1 and JNK2 were modestly regulated by NTHi infection. *Jnk1* was up-regulated for the first few days of OM, while *Jnk2* exhibited down-regulation 3–6 hours after inoculation, followed by up-regulation on subsequent days. In contrast, many of the genes up- and down-stream of JNK were strongly regulated. The up-stream JNK activator genes *Cdc42*, *Rac2* and *Arrb2*, and the down-stream target genes *cJun*, *Stat3* and *Stat4*, and the negative regulator genes *Dusp16* and *Cdc42ep2* were rapidly and persistently up-regulated. *P <0.05, **P <0.01, ***P <0.001, compared to 0 hour expression.

The expression of mRNA transcripts encoding elements of the innate immune and growth factor JNK signaling cascades leading to the activation of JNK were evaluated from ME gene array data generated before and during NTHi-induced OM. As illustrated in Figures 1, 2, 3 and Additional file 1: Table S1, genes encoding many of the elements of JNK signaling were differentially regulated during OM. Figure 1 illustrates the fold changes observed for genes that link innate immune receptors to JNK. In general, gene regulation was moderate and long-lasting. Just downstream from the receptor complex, *Traf6* and *Irak4* exhibited peaks on enhanced expression at 6 and 24 hours after inoculation, while *Tak1* exhibited more sustained up-regulation. *Jnk1* and *Jnk2* were modestly enhanced during ME infection. *Jnk3* was not only expressed at much lower initial levels than the other isoforms, but was consistently down-regulated during OM. However, the gene encoding the direct JNK phosphorylation target *Cjun* was rapidly, vigorously, and persistently up-regulated.

Figure 2 documents the regulation of many of the genes that participate in growth factor activation of JNK. Most of the significantly affected genes were up-regulated, and most peaked at 24 hours after inoculation. Exceptions were *Cdc42*, the most highly up-regulated gene, which peaked at 6 hours, and *Map3K1* and *Map3K4*, which were down-regulated in the first hours after NTHi inoculation, but then recovered to pre-inoculation levels.

Figure 3 illustrates the regulation of genes that modulate the JNK signaling pathway. All of the significantly affected positive regulators were up-regulated, most within 3 hours of inoculation. The negative regulators were more variable. *Junb* was strongly up-regulated at 3 hours and remained elevated until 2 days, while *Dusp16* peaked at 1 day. *Dusp18* was strongly down-regulated at 6 hours, while *Dusp19* was slightly up-regulated at 2 days.

While CJUN is the canonical phosphorylation target of the JNKs, many additional targets have been identified, including other potential components of the AP-1 transcriptional complex such as JUNB, JUND, and ATF2, growth regulators and apoptosis-related proteins. The expression of 38 genes encoding JNK phosphorylation targets was assessed during OM (see Additional file 2: Table S2), 18 that are activated by JNK phosphorylation, 18 that are inhibited, and 2 that can be dually affected [27]. Of these, 14 activation genes, 9 inhibition genes and both dual genes were regulated. Up-regulation was most common (20 of 25 genes), but down-regulated genes included the growth suppressor *p53* (an activation target) and the apoptosis suppressor *Bcl2* (potentially dually affected).

### Requirement of JNK1 and JNK2 for normal bacterial clearance of NTHi infection

WT mice inoculated in the ME with NTHi exhibited positive cultures at 1d, 2d and 3d, with 6/6 MEs being positive in multiple quadrants at 2d. The degree of bacterial recovery declined regularly from d1 to d3. No NTHi colonies were cultured from WT mice on d5 and thereafter. In contrast, the clearance of bacteria from JNK-deficient mice was greatly delayed. In the case of JNK1^−/−^ mice, 4/6 MEs were positive for bacteria on d5, a low degree of bacterial recovery was observed at 10d and 14d, and clearance was not achieved until 21d. In JNK2^−/−^ mice, NTHi were isolated from at least some MEs at all times, including at 21d, and the degree of bacterial colonization at later times was greater than for JNK1^−/−^ mice (Table 1).

**Table 1:** **Bacterial clearance**

**Time after NTHi inoculation**	**WT # of culture positive plates**	**WT Quadrants positive in culture positive plates**	**JNK1** ^**−/−**^ **# of culture positive plates**	**JNK1** ^**−/−**^ **Quadrants positive in culture positive plates**	**JNK2** ^**−/−**^ **# of culture positive plates**	**JNK2** ^**−/−**^ **Quadrants positive in culture positive plates**
Day 1	4/6	4.00	5/6	3.20	3/6	2.33
Day 2	6/6	3.00	2/6	3.00	4/6	2.75
Day 3	3/6	1.00	4/6	2.50	5/6	2.80
Day 5	0/6	0.00	4/6	2.00	2/6	1.50
Day 10	0/6	0.00	3/6	1.00	1/6	4.00
Day 14	0/6	0.00	2/6	1.00	1/6	1.00
Day 21	0/6	0.00	0/6	0.00	2/6	2.50

No NTHi colonies were isolated from the ME for WT mice, from 5 days after infection onward. In JNK1^−/−^ mice, NTHi were isolated up to day 14. In JNK2^−/−^ mice, NTHi was isolated throughout the 21 day observation period.

### Histology of the ME during OM

**Figure 4:**
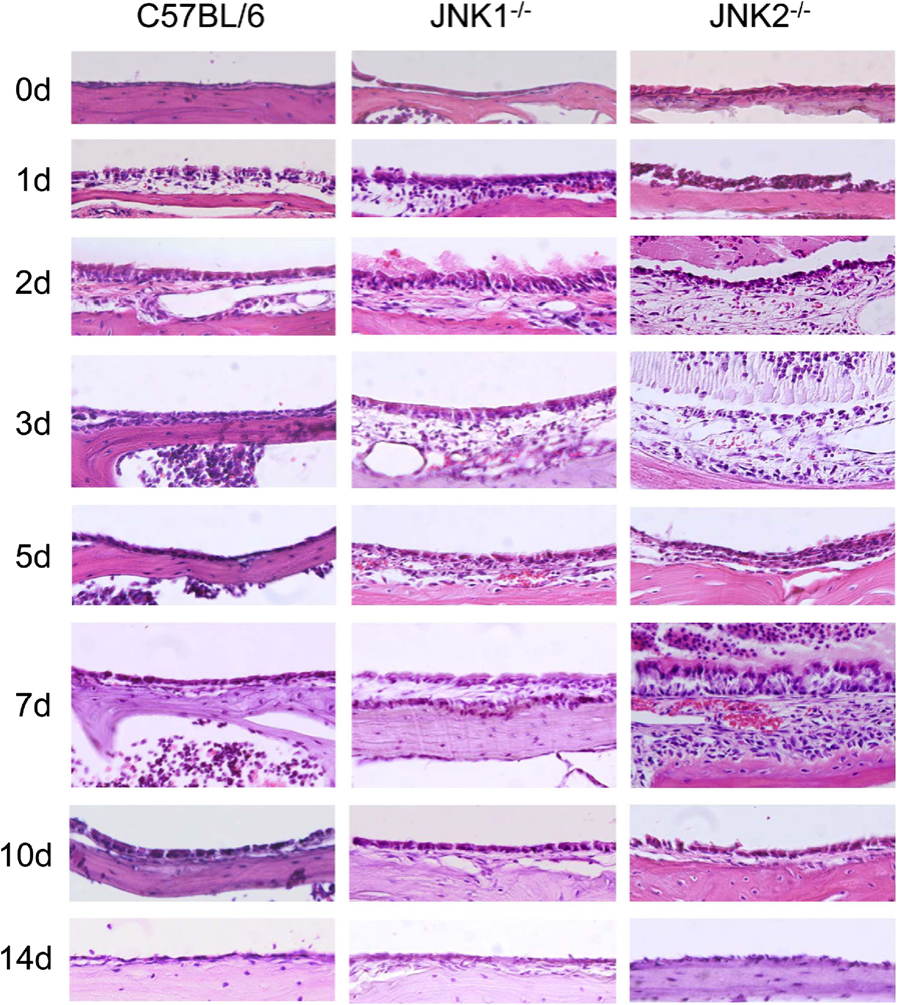
**Middle ear histology.** Representative micrographs of the ME mucosal response to nontypeable *Haemophilus influenzae* (NTHi) in C57BL/6 (WT) mice, JNK1^−/−^ mice, and JNK2^−/−^ mice. Prior to infection (0d), the MEs of WT mice have a thin, simple mucosal layer consisting of a simple, squamous epithelium and minimal stroma. In contrast, 1–2 days after infection, the mucosa significantly thickens due to expansion of the stroma and hyperplasia of the mucosa into a columnar form. From 3–5 days, the WT mucosa essentially returns to baseline condition. Prior to infection, the JNK1^−/−^ ME also exhibits a simple mucosa, which thickens considerably in the initial days after infection. However, the mucosa remains substantially hyperplasic through 7 days after infection. Prior to infection, JNK2^−/−^ MEs also have a normal-appearing mucosa, and similar to JNK1^−/−^ mice exhibit prolonged hyperplasia. However, JNK2^−/−^ mice displayed a second peak of hyperplasia 7 days after infection, prior to recovering to normal thickness by 10–14 days. Bar =50 μm.

Figure 4 illustrates the mucosal response of WT and JNK-deficient mice to NTHi inoculation of the ME. The ME of WT mice exhibit a characteristic inflammatory response when infected with NTHi [3,24,28,29]. The epithelial and stromal layers of the mucosa increase in thickness, and influxes of neutrophils and macrophages successively enter the ME cavity. In WT mice, these responses peak at d1-3 after inoculation with NTHi, and the ME returns to its pre-inoculation condition after 7–10 days. In both JNK1^−/−^ and JNK2^−/−^ mice, the ME undergoes mucosal thickening and infiltration of inflammatory cells, with the changes most prominent at d2 after inoculation. However, in JNK1^−/−^ mice the ME failed to return to a pre-inoculation condition until after 10 days. It was also observed that a subset of JNK2^−/−^ mice underwent resurgence of inflammation at d7, but also returned to normal by d10.

### Mucosal thickness

**Figure 5:**
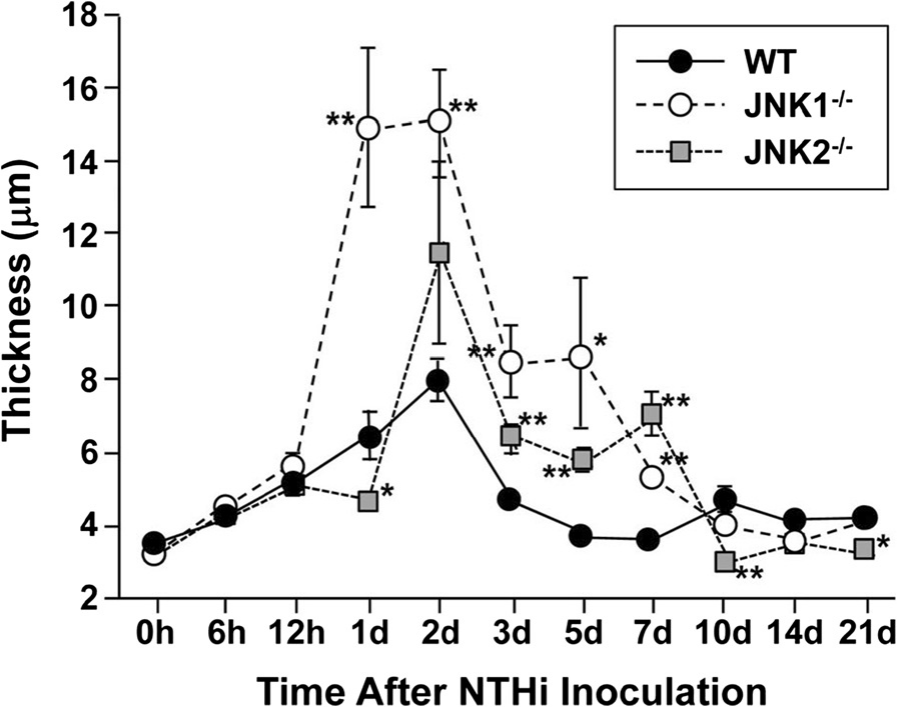
**ME mucosal hyperplasia.** Evaluation of thickness of the ME mucosal epithelium of WT (filled circles), JNK1^−/−^ (open circles) and JNK2^−/−^ (gray squares) mice after NTHi inoculation.

**Figure 6:**
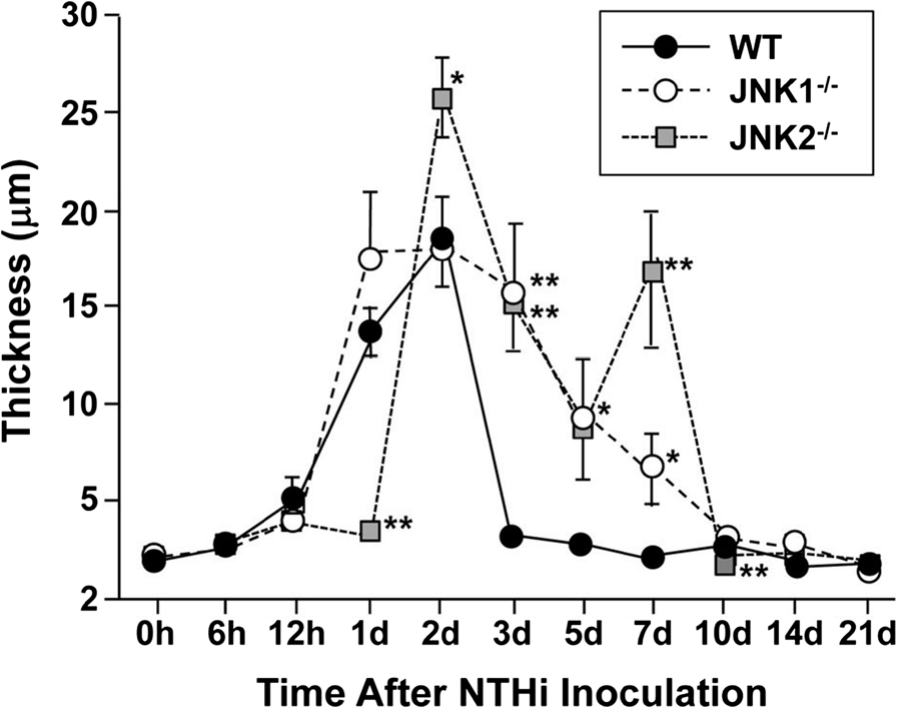
**ME mucosal hyperplasia.** Thickness of the ME subepithelial stroma. A significant delay in epithelial and stromal growth is observed for JNK2^−/−^ mice, while prolonged thickness was seen in both mutant strains. Thickness was assessed at standardized locations in the ME and averaged for each animal, and then across animals for each post-inoculation time. (* = p < .05; ** = p < .01, comparing KOs to WTs).

**Figure 7:**
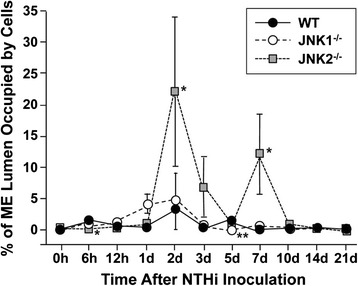
**Cellular infiltration of the ME cavity.** Percent area of the ME cavity occupied by leukocytes. Since the position of effusion in the ME is variable, the area occupied by cells was computed for the region of each ME that exhibited the maximum cellular effusion.

One measure of inflammation in the ME is the thickness of the mucosa, which displays hyperplasia in response to various inflammatory stimuli [24,29]. Quantitative data on the thickness of the ME mucosal epithelium and stroma are presented in Figures 5, 6, 7. In WT mice, modest growth of the mucosal epithelium (Figure 6) and more substantial expansion of the stroma (Figure 7) was apparent by 1d after NTHi infection. Both epithelial and stromal growth peaked at 2d and recovered baseline levels by 3-5d. In JNK1^−/−^ mice, the mucosal epithelium exhibited significant overproliferation at 1d, 2d, 3d, 5d and 7d (p ≤ .05). The stroma exhibited greater growth at 3d, 5d, and 7d (p ≤ .05). JNK2^−/−^ mice initially exhibited delayed growth of both the epithelium and stroma compared to WT mice, with significantly lower thickness at 1d (p < .05). Subsequently, the mucosal epithelium was thicker than in WT mice at 3d, 5d and 7d (p ≤ .05). The stromal compartment showed significant over proliferation at 2d, 3d and 7d (p ≤ .05). Interestingly, the epithelium and stroma of JNK2^−/−^ mice became slightly but significantly less thick than the WT at 10d (p ≤ .05).

### Leukocyte infiltration of the ME

**Figure 8:**
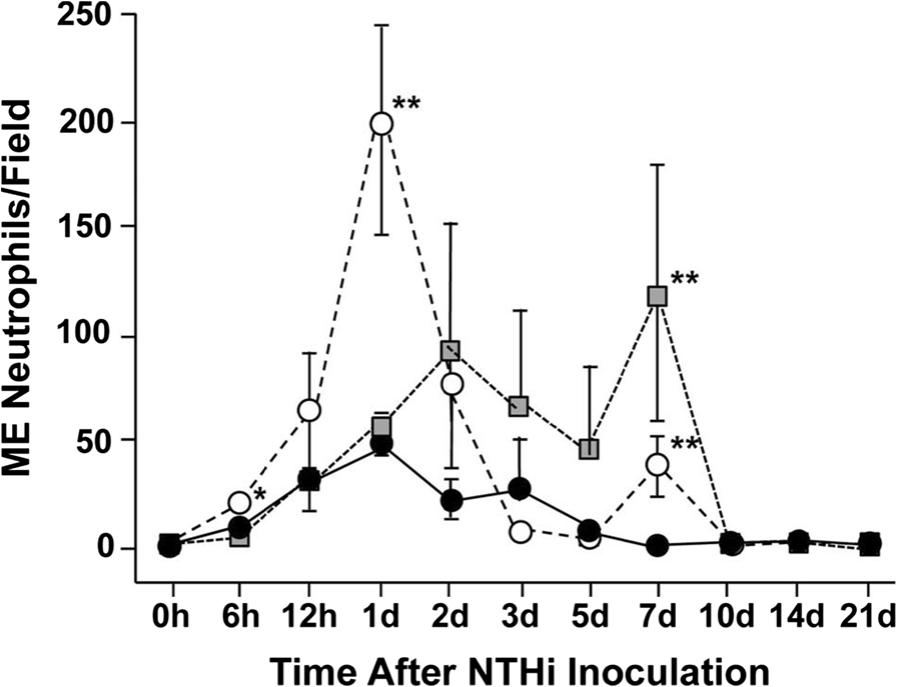
**Cellular infiltration of the ME cavity.** The number of neutrophils present in areas of cellular infiltration. A high-power (400×) field centered on the area of highest cell infiltration was imaged, and the number of neutrophils in the field was quantified.

**Figure 9:**
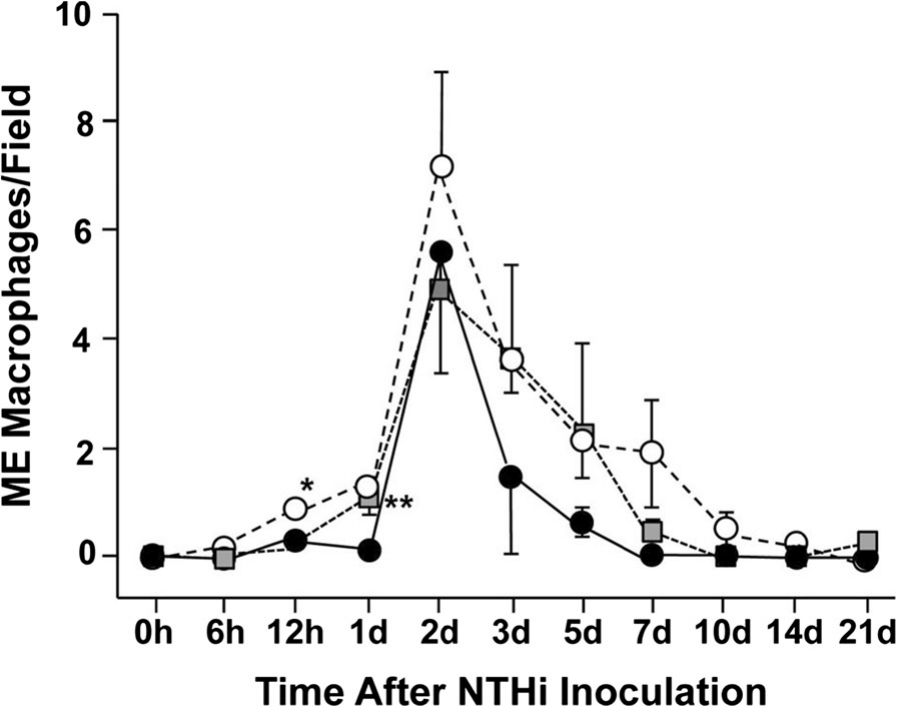
**Cellular infiltration of the ME cavity.** The number of macrophages present in areas of cellular infiltration, determined from the same images. Cell values were averaged across animals for each post-inoculation time. (* = p < .05; ** = p < .01, comparing KOs to WTs).

In WT mice, the percentage of ME cavity occupied by inflammatory cells peaked at 2d and had recovered to baseline levels by 7d (Figure 7). When infiltrating cells were further quantified as to cell type, WT mice showed neutrophil infiltration to the ME cavity beginning at 6 h, peaking at 1d, and returning to baseline levels by 5d (Figure 8). Macrophage infiltration in WTs peaked at 2d and returned to baseline levels by 7d (Figure 9).

In JNK1^−/−^ mice, the percentage of the ME occupied by inflammatory cells was similar to that observed in WTs, with the exception that a somewhat lower proportion of cells was seen at 5d (p ≤ .01) (Figure 7). When cell infiltration was evaluated for cell type, JNK1^−/−^ neutrophil infiltration slightly but significantly exceeded that seen in WTs at 6 h (p ≤ .05), was dramatically higher at 1d and modestly higher at 7d (p ≤ .01) (Figure 8).

JNK2^−/−^ mice showed substantially greater inflammatory cell infiltration into the ME cavity at 2d and 7d (p ≤ .05), and a slight but significant decrease at 6 h (Figure 7). JNK2^−/−^ neutrophil infiltration was significantly increased at 7d (p ≤ .01) (Figure 8), while macrophage infiltration was dramatically increased at 2d (p ≤ .01) (Figure 9).

## Discussion

### Regulation of JNK signaling genes supports a role in the host response to ME infection

As seen in the microarray data for the ME mucosa, many of the genes that are involved in the JNK pathway show significant regulation during the course of OM. This degree of regulation suggests that the pathway plays a critical role in this condition. It is significant that the up-regulation of most genes peaked at 1d after inoculation. ME inflammation, leukocytic infiltration and mucosal hyperplasia all peaked at 2d after inoculation. Given the delays to be expected in protein synthesis and cellular responses, the increase in gene expression related to JNK signaling is well positioned to participate in all three manifestations of OM.

It should be noted that we observed only modest regulation of the genes encoding JNK1 and JNK2 themselves. This suggests that these signaling molecules are maintained at functional levels in the resting ME, and are predominantly regulated post-transcriptionally, perhaps by phosphorylative activation. This is consistent with prior data at the protein level demonstrating little change in total JNK during OM, but increased phospho-JNK1 and -JNK2 [23]. In contrast, the genes encoding signaling molecules upstream from JNK (e.g. IRAK4, CDC42, KRAS) were up-regulated during OM, suggesting increased involvement of JNK signaling during the course of OM. Again, protein data confirms a peak of phospho-JNK at 72 h after initiation of infection [23]. We also found that genes encoding both positive (e.g. ARRB2, RAC2) and negative (e.g. CDC42EP2, DUSP16) regulators of the JNK pathway showed significant up-regulation during OM, suggesting tight regulation of JNK activity, perhaps to prevent bystander injury to host tissues from inflammation and/or apoptosis [30].

The gene encoding the primary downstream target of JNK, CJUN, showed very strong and early up-regulation. CJUN dimerizes with members of the JUN, FOS or ATF2 subfamilies to create AP-1 [31], a multifunctional transcription factor. AP-1 induces the production of multiple pro-inflammatory cytokines, including tumor necrosis factor alpha (TNFA) [32]. TNFA is a proinflammatory mediator important in innate immunity. It is strongly expressed during OM and TNFA^−/−^ mice have shown delayed NTHi-induced OM resolution [28]. This suggests that the JNK pathway is critical in inducing inflammation, which in turn is critical for proper recruitment and access of immune cells to the site of infection for infection clearance. This is consistent with the delayed bacterial clearance observed in both JNK1- and JNK2-deficient mice. AP-1 is also implicated in tissue proliferation [33]. Its primary components, CJUN and CFOS, are both positive regulators of tissue growth and their active heterodimers can cause anchorage-independent proliferation [34]. The up-regulation of GF-related JNK signaling genes is consistent with a role for JNK in tissue proliferation during OM. The regulation of a majority of genes encoding direct JNK phosphorylation targets involved in AP-1 transcription, tissue proliferation and apoptosis [11,35,36] further implicates the molecules of the JNK signaling system in OM.

### JNK deletion indicates that both isoforms play a role in OM

Both JNK1^−/−^ and JNK2^−/−^ mice showed significantly different physiological responses to NTHi infection of the ME when compared to WT animals. Both mutants exhibited delayed bacterial clearance, as well as atypical leukocyte recruitment. Both JNK1^−/−^ and JNK2^−/−^ also showed a delayed return to baseline mucosal thickness. Interestingly, both JNK1^−/−^ and JNK2^−/−^ MEs showed evidence of a resurgence of OM at 7d, after the initiation of recovery. The similarity of these changes suggests that JNK1 and JNK2 are both necessary for a normal innate immune response to OM, and that some functions of the two isoforms appear to overlap.

Albeit delayed, eventual resolution of infection was observed in all JNK1^−/−^ mice and in most JNK2^−/−^ mice. Moreover, the basic histopathologic features of OM, although altered, were present in both strains, and both mutants showed return of mucosal thickness and leukocyte levels to baseline. This suggests redundancy between the two isoforms and/or JNK-independent mechanisms that operate in parallel with JNK-dependent processes to mediate pathogenesis and recovery from OM. NFκB, known to mediate innate immune responses in addition to JNK, is a strong candidate for such a parallel pathway [37].

We did not obtain evidence that an individual JNK isoform is responsible for hyperplasia of the ME mucosa, since neither JNK1- nor JNK2-deficient animals exhibited a consistently decreased proliferative response to bacterial infection. Rather, the proliferative response was more persistent, presumably reflecting prolonged bacterial infection. Since we have previously shown that a pan-JNK inhibitor reduces mucosal proliferation during OM [23], our current results suggest redundancy between the two isoforms. Of course, JNK-independent pathways may also be involved. Growth factor stimulation can activate alternative growth pathways mediated by the extracellular signal-regulated kinases (ERK) and p38 MAP kinases, both of which have been shown to be activated during OM [38,39], and as above NFκB is another potential contributory pathway.

### JNK1 and JNK2 have distinct roles in regulating the ME response to NTHi

Although the mechanistic differences remain unclear, it is known that JNK1 and JNK2 are not necessarily interchangeable in the JNK signaling pathway. This is also apparent in our data, since the two isoforms were unable to completely compensate for each other during OM and since the ME phenotypes of the two mutants were distinct. JNK1-deficient mice exhibited earlier growth of the mucosal epithelium compared to the WT. In contrast, JNK2^−/−^ mice exhibited a delay in the growth of both the mucosal epithelium and stroma. JNK1^−/−^ mice exhibited greatly enhanced neutrophil recruitment early in OM, while JNK2-deficient mice exhibited a delay in neutrophil recruitment and greater persistence of neutrophils. JNK2^−/−^ mice also showed enhanced macrophage recruitment to the ME, while JNK1^−/−^ mice did not. The phenotype of JNK2^−/−^ mice suggests a more serious defect in inflammation than seen in JNK1^−/−^ mice, given the delays in mucosal proliferation and leukocyte recruitment and the greater persistence of bacterial infection in these mice. This may reflect the reported higher levels of phosphorylated JNK2 detected in the ME after bacterial inoculation, when compared to JNK1 [23].

The fact that JNK1-deficient mice exhibit enhanced neutrophil recruitment and epithelial thickness early in OM could reflect a role for this isoform in limiting inflammation during early OM. Supporting this possibility, JNK1 has been found to be protective against ischemic injury in the brain [40].

### Implications and limitations of the study

Drawing conclusions from animal studies regarding human disease must, of course, be performed with caution. Although the characteristics of NTHi-induced OM in mice are quite similar to those observed in humans (e.g. [3,29]), differences in genetics and in the response of mice to a human pathogen must be considered in the interpretation of data. With this caveat, our findings have implications for the treatment of OM. The use of JNK inhibitors alone to treat OM would probably not be advisable, given the possibility of reduced bacterial clearance. However, our data suggest that, in combination with antibiotics, JNK inhibition has the potential to reduce inflammation and mucosal hyperplasia. Any such strategy would need to take into account differences the roles of JNK1 and JNK2 in OM. Thus, a JNK2-specific inhibitor might be more beneficial than a pan-JNK inhibitor.

## Conclusions

In summary, we found that JNK signaling plays a significant role in OM, as reflected by altered expression of JNK signaling genes, as well as reduced bacterial clearance and altered ME phenotype in mice deficient in JNK isoforms. JNK1 and JNK2 are both required for the normal resolution of NTHi infection in the ME. JNK1 and JNK2 appear to play both redundant and distinct roles in OM, with JNK2 mediating the early recruitment of neutrophils and being more critical for bacterial clearance. JNK1 may actually limit inflammation early in OM. The results have implications for JNK inhibition as a therapeutic option in OM.

## Additional files

Additional file 1: Table S1. Change in expression of JNK signaling genes during acute OM. Additional file 2: Table S2. Change in expression of JNK target genes during acute OM.

## Abbreviations

CSOM: Chronic suppurative OM
DUSP: Dual specificity phosphatase
ERK: Extracellular signal-regulated kinases
GO: Gene ontogeny
JIPs: JNK-interacting proteins
JNK: C-Jun N-terminal Kinase
MAP2Ks: MAP kinase kinases
MAP3K: MAPK kinase kinase kinases
MAPKs: Mitogen activated protein kinases
ME: Middle ear
NTHi: Nontypeable *Haemophilus influenzae*
OM: Otitis media
POSH: Plenty of SH3
TLR: Toll-like receptor
TNFA: Tumor necrosis factor alpha
VAMPIRE: Variance-modeled posterior inference
WT: Wildtype
