# Supplementary material for: C-Jun N-terminal kinase (JNK) isoforms play differing roles in otitis media
Source: BMC Immunol. 2014 Oct 14;15:46. doi: 10.1186/s12865-014-0046-z (PMC4200133; doi:10.1186/s12865-014-0046-z)
Supplement: Additional file 1: Table S1. — Change in expression of JNK signaling genes during acute OM. [file 12865_2014_46_MOESM1_ESM.doc]

**Additional file 1: TableS1. Change in Expression of JNK Signaling Genes During Acute OM.**

***1. TLR JNK Signaling Genes***

**Time: 0h 3h 6h 24h 2d 3d 5d 7d**

***Irak4*** (1421670_a_at)

Fold Exp 0.9 1.4 0.6 3.8 1.4 0.8 1.1 2.0

Range 0.5–1.5 1.4-1.5 0.2-1.4 3.6-3.9 0.6-3.6 0.2-2.8 0.6-1.9 1.6-2.4

P-Value 0.84 0.07 0.65 **0.02** 0.76 0.89 0.89 0.93

***Traf6*** (1435350_at)

Fold Exp 1.0 2.0 4.1 1.6 1.8 1.5 0.6 1.2

Range 0.7-1.3 1.9-2.1 2.9-5.7 1.5-1.7 1.7-2.0 1.2-1.8 0.6-0.6 1.2-1.2

P-Value 0.91 **0.03** 0.15 0.11 0.08 0.28 **0.03** **0.00**

***Tak1*** (1426627_at)

Fold Exp 0.99 1.6 1.6 3.1 2.4 2.2 2.5 1.8

Range 0.8-1.2 1.6-1.6 1.5-1.7 2.3-4.1 2.3-2.4 2.0-2.4 2.5-2.6 1.8-1.9

P-Value 0.95 **0.03** 0.11 0.16 **0.02** 0.07 **0.01** **0.03**

***Jnk1*** (420932_at)

Fold Exp 1.0 1.2 1.4 1.6 1.3 1.4 0.9 1.1

Range 0.9-1.1 1.2-1.2 1.2-1.7 1.6-1.7 1.2-1.3 1.2-1.6 0.9-0.9 1.0-1.2

P-Value 0.97 **0.02** 0.25 **0.04** 0.18 0.29 0.16 0.53

***Jnk2*** (1421876_at)

Fold Exp 1.0 0.6 0.5 1.5 1.6 1.6 1.7 1.5

Range 0.9-1.1 0.5-0.7 0.5-0.5 1.5-1.5 1.5-1.8 1.5-1.6 1.6-1.7 1.3-1.8

P-Value 0.97 0.26 0.06 **0.004** 0.14 **0.03 0.03** 0.20

***Jnk3*** (1440201_at)

Fold Exp 1.0 0.4 0.3 0.3 0.4 0.8 0.7 0.6

Range 1.0-1.0 0.3-0.4 0.2-0.5 0.2-0.3 0.3-0.5 0.8-0.9 0.6-0.7 0.6-0.7

P-Value 0.99 0.08 0.17 **0.02** 0.18 0.29 0.10 0.15

***cJun*** (1448694_at)

Fold Exp 1.0 10.3 6.9 9.1 5.8 2.8 1.4 1.1

Range 0.9-1.1 9.1-11.6 6.8-6.9 8.6-9.6 5.6-6.1 2.1-3.7 1.3-1.4 1.0-1.1

P-Value 0.98 **0.03** **0.001** **0.02** **0.01** 0.17 0.12 0.11

***2. GF JNK Signaling Genes***

**Time: 0h 3h 6h 24h 2d 3d 5d 7d**

***Shc1*** (1422853_at)

Fold Exp 0.9 1.4 1.5 2.5 1.7 1.4 1.7 1.7

Range 0.7-1.3 1.2-1.7 1.3-1.8 2.4-2.7 1.7-1.8 1.1-1.8 1.4-2.1 1.4-2.0

P-Value 0.89 0.32 0.21 **0.05 0.04** 0.43 0.23 0.22

***Grb2*** (1449111_a_at)

Fold Exp 1.0 2.0 1.7 4.6 3.4 2.4 1.6 1.0

Range 1.0-1.0 1.6-2.3 1.6-1.8 4.4-4.8 2.7-4.2 1.9-2.8 1.5-1.6 0.9-1.2

P-Value 0.99 0.16 0.07 **0.02** 0.11 0.14 0.06 0.78

***Sos1*** (1421886_at)

Fold Exp 1.0 1.6 2.0 2.1 1.8 2.5 0.8 1.2

Range 0.8-1.2 0.9-3.1 2.0-2.1 1.6-2.7 1.8-1.8 2.4-2.7 0.5-1.3 0.7-2.1

P-Value 0.94 0.58 **0.02** 0.22 **0.02 0.04** 0.75 0.79

***Kras*** (1451979_at)

Fold Exp 1.0 2.1 2.2 3.1 2.2 1.5 1.2 1.0

Range 0.9-1.1 2.0-2.2 1.9-2.5 3.1-3.1 2.1-2.3 1.5-1.5 1.1-1.2 0.9-1.1

P-Value 0.96 **0.05** 0.10 **0.003 0.05 0.03** 0.12 0.85

***Cdc42*** (1435807_at)

Fold Exp 0.7 5.9 9.9 7.0 6.5 4.1 3.5 3.8

Range 0.3-1.7 5.9-6.0 8.7-11.3 5.7-8.4 5.4-7.9 3.3-5.1 3.3-3.8 3.7-3.9

P-Value 0.77 **0.003 0.04** 0.06 0.06 0.10 **0.04 0.02**

***Mlk3*** (1450669_at)

Fold Exp 1.0 2.1 1.6 4.1 2.9 0.6 0.2 0.1

Range 0.9-1.1 2.0-2.1 1.4-1.8 3.6-4.7 2.5-3.5 0.2-1.7 0.2-0.2 0.1-0.2

P-Value 0.97 **0.03** 0.18 0.06 0.10 0.71 **0.05 0.05**

***2. GF JNK Signaling Genes* (continued)**

**Time: 0h 3h 6h 24h 2d 3d 5d 7d**

***Map3K1*** (1424850_at)

Fold Exp 1.0 **0.5 0.4** 0.6 1.3 0.8 1.0 0.8

Range 0.9 – 1.2 0.4 – 0.5 0.3 – 0.4 0.6 – 0.7 0.8 – 2.0 0.8 – 0.8 0.8 – 1.1 0.8 – 0.9

P-Value 0.95 0.12 **0.04 0.05** 0.69 0.07 0.87 0.27

***Map3K4*** (1421450_a_at)

Fold Exp 1.0 0.6 **0.4** 0.9 1.0 0.9 0.8 0.8

Range 1.0 – 1.0 0.6 – 0.6 0.3 – 0.5 0.9 – 1.0 0.9 – 1.0 0.9 – 1.0 0.7 – 0.9 0.7 – 1.0

P-Value 0.99 **0.02** 0.18 0.61 0.71 0.43 0.24 0.46

***Map2K7*** (1425513_at)

Fold Exp 1.0 1.1 0.9 2.6 1.6 1.7 1.0 1.2

Range 0.7-1.3 1.1-1.2 0.7-1.2 1.4-4.8 1.6+1.6 1.5-1.8 1.0-1.0 1.1-1.2

P-Value 0.91 0.13 0.80 0.38 0.006 0.10 0.99 0.15

***Cdc42ep2*** (1428750_at)

Fold Exp 1.0 3.0 2.9 3.8 2.3 1.3 1.0 1.2

Range 1.0-1.0 3.0-3.1 2.1-4.0 3.6-4.0 2.2-2.4 1.2-1.5 1.0-1.0 1.2-1.2

P-Value 0.99 **0.01** 0.18 **0.02 0.03** 0.26 0.90 **0.02**

***3. JNK Regulatory Genes***

**Time: 0h 3h 6h 24h 2d 3d 5d 7d**

***Arrb2*** (1451987_at)

Fold Exp 0.9 1.8 1.9 10.4 8.4 3.6 1.8 1.6

Range 0.6-1.4 1.7-1.9 1.6-2.1 10.2-10.5 7.8-8.9 2.6-5.0 1.4-2.3 1.5-1.7

P-Value 0.88 0.06 0.14 **0.004 0.02** 0.16 0.24 0.06

***Dusp1*** (1448830_at)

Fold Exp 1.0 13.6 12.7 12.4 7.7 3.8 0.8 1.4

Range 0.9-1.1 13.5-13.7 12.3-13.1 11.3-13.6 6.8-8.8 3.3-4.4 0.8-0.9 0.7-2.6

P-Value 0.96 **0.002 0.008** 0.22 **0.04** 0.07 0.31 0.69

***Dusp2*** (1450698_at)

Fold Exp 1.0 5.8 4.5 2.0 1.0 0.6 0.7 0.7

Range 0.9-1.1 5.2-6.4 4.0-5.0 1.9-2.1 0.8-1.2 0.3-1.0 0.7-0.7 0.7-0.7

P-Value 0.98 **0.04 0.04 0.04** 0.97 0.49 0.10 **0.01**

***Dusp8*** (1418714_at)

Fold Exp 1.0 5.2 3.4 2.3 1.3 1.3 1.1 1.2

Range 0.9-1.1 3.7-7.1 3.3-3.6 2.2-2.4 1.1-1.5 0.8-2.1 0.9-1.3 1.0-1.5

P-Value 0.98 0.13 **0.02 0.04** 0.32 0.69 0.82 0.52

***Dusp16*** (1418401_a_at)

Fold Exp 1.0 4.2 4.1 8.0 2.6 1.4 0.8 0.6

Range 0.8-1.2 3.7-4.7 3.7-4.6 7.9-8.0 2.3-2.9 1.3-1.5 0.7-0.8 0.6-0.7

P-Value 0.95 0.05 0.05 **0.002** 0.07 0.11 0.14 0.5

***Dusp18*** (1437866_at)

Fold Exp 1.0 0.5 0.3 0.9 1.1 0.7 0.8 1.0

Range 0.9-1.1 0.5-0.6 0.3-0.3 0.8-1.1 1.0-1.2 0.6-0.8 0.7-1.0 0.9-1.2

P-Value 0.96 0.08 0.04 0.74 0.52 0.29 0.48 0.88

***Dusp19*** (1418038_s_at)

Fold Exp 1.0 0.4 0.4 1.5 1.7 1.5 1.2 1.3

Range 0.9-1.1 0.3-0.5 0.4-0.5 1.3-1.7 1.7-1.8 1.3-1.7 1.1-1.2 1.2-1.4

P-Value 0.98 0.12 0.14 0.23 **0.01** 0.19 0.15 0.23

***Junb*** (1415899_at)

Fold Exp 0.9 13.5 11.4 12.7 4.0 1.8 1.1 1.4

Range 0.6-1.4 12.6-14.4 11.2-11.5 11.9-13.5 3.8-4.2 1.1-2.8 1.0-1.2 1.3-1.6

P-Value 0.86 **0.02** 0.004 **0.02** **0.02** 0.42 0.32 0.15
