# Supplementary material for: C-Jun N-terminal kinase (JNK) isoforms play differing roles in otitis media
Source: BMC Immunol. 2014 Oct 14;15:46. doi: 10.1186/s12865-014-0046-z (PMC4200133; doi:10.1186/s12865-014-0046-z)
Supplement: Additional file 2: Table S2. — Change in expression of JNK target genes during acute OM. [file 12865_2014_46_MOESM2_ESM.doc]

# Additional file 2: TableS2. Change in Expression of JNK Target Genes During Acute OM.

***1. JNK Activation Targets***

# Time: 0h 3h 6h 24h 2d 3d 5d 7d

***Jund*** (1440265_at)

Fold Exp 1.0 1.5 0.9 2.3 1.9 1.4 0.8 2.1

Range 0.7 – 1.3 1.1 – 2.1 0.7 – 1.0 2.2 – 2.3 1.4 – 2.6 0.7 – 3.1 0.7 – 0.9 1.2 – 3.7

P-Value 0.91 0.41 0.52 **0.02** 0.28 0.72 0.28 0.40

***ATF2*** (1426583_at)

Fold Exp 1.0 0.6 0.5 0.9 1.0 1.0 1.0 0.9

Range 0.9 – 1.1 0.6 – 0.6 0.4 – 0.5 0.9 – 1.0 0.8 – 1.2 0.9 – 1.2 1.0 – 1.0 0.9 – 1.0

P-Value

***Myc*** (1424942_a_at)

Fold Exp 0.9 5.5 5.1 4.0 1.4 1.6 0.8 0.7

Range 0.7 – 1.3 5.4 – 5.6 4.9 – 5.3 3.3 – 4.9 1.2 – 1.6 1.4 – 1.8 0.8 – 0.9 0.5 – 0.8

P-Value 0.90 **0.00** **0.02** 0.09 0.26 0.17 0.20 0.26

***P53 (Trp53)*** (1457623_x_at)

Fold Exp 1.0 0.8 0.5 0.5 1.0 0.0 0.5 0.7

Range 0.7 – 1.3 0.8 – 0.8 0.4 – 0.7 0.5 – 0.6 0.7 – 1.3 0.04 – 0.06 0.4 – 0.5 0.7 – 0.7

P-Value 0.92 **0.00** 0.26 0.09 0.95 **0.04** **0.05** **0.01**

***Foxo4 (Mllt7)*** (1422149_at)

Fold Exp 0.9 2.2 2.0 1.0 0.3 0.4 0.8 1.6

Range 0.5 – 1.5 2.1 – 2.2 1.9 – 2.1 0.9 – 1.0 0.1 – 1.2 0.4 – 0.4 0.8 – 0.9 1.2 – 2.3

P-Value 0.85 **0.02** **0.04** 0.88 0.54 **0.04** 0.29 0.38

***Stat3*** (1426587_a_at)

Fold Exp 0.8 4.7 4.7 5.9 5.2 2.8 2.1 2.1

Range 0.4 – 1.6 3.4 – 6.4 4.2 – 5.2 5.8 – 6.0 4.6 - -5.9 2.7 – 2.9 1.9 – 2.3 1.9 – 2.3

P-Value 0.79 0.13 **0.04** **0.01** **0.05** **0.03** 0.08 0.08

***Bad*** (1416583_at)

Fold Exp 1.0 1.0 0.7 2.8 2.1 1.5 1.3 1.4

Range 0.7 – 1.3 1.0 – 1.1 0.6 – 0.8 2.4 – 3.4 2.0 - 2.2 1.3 – 1.8 1.2 – 1.4 1.3 – 1.5

P-Value 0.91 0.28 0.29 0.11 **0.03** 0.25 0.22 0.09

***Bax*** (1416837_at)

Fold Exp 1.0 1.4 1.4 4.1 2.8 2.4 1.3 1.4

Range 0.9 – 1.1 1.4 – 1.4 1.2 – 1.6 4.0 – 4.2 2.5 – 3.2 2.1 – 2.8 1.3 – 1.4 1.3 – 1.5

P-Value 0.97 **0.04** 0.29 **0.01** 0.07 0.10 **0.05** 0.16

***Bim* (Bcl2l11)** (1426334_a_at)

Fold Exp 1.0 2.0 2.1 5.7 4.0 2.9 0.8 1.2

Range 0.9 – 1.1 1.9 – 2.0 1.7 – 2.6 5.0 – 6.5 3.6 – 4.4 2.0 – 4.2 1.4 – 2.2 1.1 – 1.3

P-Value 0.97 **0.03** 0.18 **0.05** **0.05** 0.21 0.22 0.30

***Pxn (paxillin)*** (1456135_s_at)

Fold Exp 1.0 1.5 1.1 2.5 1.7 1.0 0.7 0.6

Range 1.0 – 1.0 1.4 – 1.7 1.0 – 1.2 2.5 – 2.6 1.7 – 1.7 0.8 – 1.3 0.7 – 0.7 0.6 – 0.6

P-Value 0.99 0.14 0.45 **0.01** **0.02** 0.94 0.09 **0.03**

***2. JNK Inhibition Targets***

# Time: 0h 3h 6h 24h 2d 3d 5d 7d

***Nfatc1*** (1425761_a_at)

Fold Exp 0.9 3.1 3.0 3.0 1.9 1.4 1.4 1.9

Range 0.6 – 1.4 3.0 – 3.2 2.7 – 4.0 2.5 – 3.6 1.5 – 2.4 1.3 – 1.6 1.3 – 1.5 1.7 – 2.1

P-Value 0.88 **0.02** 0.07 0.11 0.22 0.12 0.18 0.09

***Nfatc3*** (1452497_a_at)

Fold Exp 0.6 1.2 1.2 4.1 3.4 2.8 3.5 3.8

Range 0.2 – 1.8 1.2 – 1.3 0.9 – 1.5 3.8 – 4.5 2.7 – 4.3 2.7 – 3.0 3.4 – 3.5 3.7 – 3.9

P-Value 0.71 0.08 0.67 0.04 0.12 **0.03** **0.01** **0.01**

***Nur77*** (Nr4a1 1416505_at)

Fold Exp 1.0 11.0 5.8 3.6 2.0 1.6 0.8 1.1

Range 0.9 – 1.1 9.5 – 12.7 5.3 – 6.4 3.3 – 3.9 1.7 – 2.5 1.4 – 1.8 0.7 – 1.0 0.7 – 1.4

P-Value 0.97 **0.04** **0.04** **0.04** 0.17 0.16 0.54 0.83

***2. JNK Inhibition Targets* (continued)**

# Time: 0h 3h 6h 24h 2d 3d 5d 7d

***Akt1*** (1425711_a_at)

Fold Exp 0.4 2.9 2.6 9.1 4.1 2.3 1.9 1.9

Range 0.1 – 1.9 2.8 – 3.1 2.4 – 2.7 8.6 – 9.6 3.1 – 5.4 2.1 – 2.4 1.8 – 2.1 1.6 – 2.2

P-Value 0.65 **0.03** **0.04** **0.02** 0.12 0.06 0.08 0.15

***Akt3*** (1460307_at)

Fold Exp 1.0 0.8 0.5 2.6 3.1 2.2 1.4 0.9

Range 0.8 – 1.3 0.7 – 1.0 0.3 – 0.8 2.3 – 2.9 3.0 – 3.1 1.9 – 2.6 1.3 – 1.5 0.4 – 2.0

P-Value 0.92 0.41 0.35 0.08 **0.01** 0.12 0.15 0.92

***Ets1*** 1 (1422028_a_at)

Fold Exp 1.0 2.3 2.1 1.5 1.1 0.8 0.9 0.9

Range 0.8 – 1.2 2.3 – 2.4 2.0 – 2.3 1.4 – 1.6 0.8 – 1.7 0.6 – 1.1 0.8 – 1.0 0.8 – 0.9

P-Value 0.95 **0.00** 0.07 0.09 0.81 0.57 0.48 0.15

***Bclxl (Bcl2l1)*** (1426334_a_at)

Fold Exp 0.4 2.7 3.0 3.3 2.2 0.5 1.4 1.3

Range 0.1 – 1.9 2.3 – 3.2 2.8 – 3.1 3.0 – 3.6 1.9 – 2.5 0.4 – 0.8 1.4 – 1.4 1.2 – 1.3

P-Value 0.66 0.11 **0.03** **0.05** 0.12 0.35 **0.04** 0.16

***Tau (Mapt)*** (1455028_at)

Fold Exp 1.0 0.7 0.6 0.4 0.5 0.8 0.7 0.8

Range 0.8 – 1.2 0.7 – 0.7 0.6 – 0.7 0.3 – 0.4 0.4 – 0.5 0.7 – 0.8 0.6 – 0.9 0.7 – 0.8

P-Value 0.93 0.09 0.08 **0.01** **0.03** 0.12 0.44 0.20

***Mcl1*** (1416881_at)

Fold Exp 1.0 8.7 9.0 14.3 7.1 3.7 1.9 1.8

Range 0.9 – 1.1 8.0 – 9.3 8.9 – 9.0 12.5 – 16.3 6.4 – 8.0 3.1 – 4.3 1.9 – 1.9 1.7 – 1.8

P-Value 0.97 **0.02** **0.00** **0.03** 0.03 0.08 **0.00** **0.02**

***3. JNK Dually Regulated Targets***

# Time: 0h 3h 6h 24h 2d 3d 5d 7d

***Stat4*** (1448713_at)

Fold Exp 1.0 15.0 18.1 30.1 19.5 19.0 0.9 11.7

Range 0.9 – 1.1 12.4 – 20.0 12.1 – 26.9 30.0 – 30.3 14.5 – 26.4 13.7 – 26.2 0.8 – 0.9 10.0 – 13.6

P-Value 0.97 0.06 0.09 **0.00** 0.06 0.07 0.23 **0.04**

***Ddit3*** (1417516_at)

Fold Exp 1.0 1.2 1.2 4.9 2.5 3.9 1.7 1.6

Range 0.9 – 1.1 1.1 – 1.4 0.9 – 1.6 4.7 – 5.0 1.7 – 3.8 3.5 – 4.3 1.6 – 1.7 1.4 – 2.0

P-Value 0.96 0.37 0.64 **0.01** 0.26 0.05 0.05 0.23

***Bcl2*** (1431122_at)

Fold Exp 1.0 0.6 0.6 0.5 0.4 0.3 0.4 0.7

Range 0.8 – 1.2 0.6 – 0.7 0.5 – 0.6 0.4 – 0.7 0.3 – 0.4 0.3 – 0.4 0.4 – 0.4 0.7 – 0.7

P-Value 0.93 0.17 0.12 0.22 0.12 0.14 **0.02** **0.01**
